# Review: Efficacy of Neuroimaging Methods on the Prediction of Conversion from MCI to AD in a Clinical Setting

**DOI:** 10.1002/alz70858_096518

**Published:** 2025-12-24

**Authors:** Stephanie Li

**Affiliations:** ^1^ Boston University Chobanian & Avedisian School of Medicine, Boston, MA, USA

## Abstract

**Background:**

This systemic review bridges any gaps in current literature and knowledge regarding the efficacy of neuroimaging modalities in the prediction of the conversion of MCI (mild cognitive impairment) to AD (Alzheimer's disease) dementia, and provides a recommendation for the best neuroimaging

**Method:**

A comprehensive literature review was performed using SCOPUS, Google Scholar, and PubMed with the combination of any of the following keywords: “neuroimaging,” “MCI to AD,” “conversion,” “prediction,” “MRI,” “FDG‐PET,” “SPECT,” “EEG,” and Research Rabbit was used to discover relevant literature based on previously searched papers. Paper exclusion criteria included: those without a specific mention of MCI‐AD conversion in their titles, ones that discussed the conversion of MCI to any other form of dementia besides AD dementia, papers with inconclusive evidence of efficacy, papers that did not use neuroimaging methods, and any papers that mentioned “MCI due to AD.” Additionally, papers were only considered if they were published between the years of 2000 to 2024.

**Result:**

A total of 89 papers were reviewed for this review paper, and 27 papers were excluded from the final review due to meeting one or more of the exclusion criteria. There were 6 total papers for EEG studies, 16 papers for SPECT, 31 for MRI studies, and 7 for FDG‐PET.

**Conclusion:**

In a clinical setting, there may be limited resources and funding available for such predictive methods, so while the usage of SPECT or FDG‐PET may be effective methods in the prediction of MCI‐AD conversion in laboratory settings, they require resources that most clinics do not have and utilize radioactive tracers. While EEG is a promising up‐and‐coming methodology, more research needs to be done on its efficacy in a clinical setting. Based on the current evidence available, MRI (specifically sMRI and DTI) has the highest predictive ability of MCI‐AD conversion in a clinical setting.

